# Dynamic changes in myeloid-derived suppressor cells during the menstrual cycle: A pilot study

**DOI:** 10.3389/fmed.2022.940554

**Published:** 2022-11-15

**Authors:** Qiying Xu, Huifang Liu, Muge Qile, Tana Wuren

**Affiliations:** ^1^Department of Gynecology, Affiliated Hospital of Qinghai University, Xining, China; ^2^Key Laboratory for Application of High-Altitude Medicine, Qinghai University, Xining, China; ^3^Research Center for High Altitude Medicine, Qinghai University, Xining, China

**Keywords:** myeloid-derived suppressor cells, physiological conditions, menstrual cycle, estradiol (E2), prostaglandin E2 (PGE2)

## Abstract

Various studies have described the roles of myeloid-derived suppressor cells (MDSCs) in pathological conditions, but relatively few have described them under normal physiological conditions. Accumulation of MDSCs is important creating an anti-inflammation environment, which is essential for fertilized egg implantation. This study was designed to record the dynamic changes in MDSC-like cells composition during the menstrual period (MP) and ovulation period (OP) in healthy volunteers over the course of a single menstrual cycle to explore the association between MDSCs and the menstrual cycle under normal physiological conditions. The ratio of MDSC-like cells was higher in MP samples, whereas the activity of Arg-1 was higher during the OP window. There was a negative correlation between the ratio of MDSC-like cells and the percentage of lymphocytes and a positive correlation between MDSC-like cells and prostaglandin E2 (PGE2). Furthermore, regular changes in the ratio and function of MDSC-like cells in the peripheral blood were observed during menstruation, all of which corresponded to the cycle stage. During menstruation, MDSCs may promote endometrial repair, whereas they promote pregnancy during the OP. These findings may help to better understand the pathophysiology of pregnancy-related complications and lay a foundation for improving perinatal outcomes.

## Introduction

Myeloid-derived suppressor cells (MDSCs) were originally identified as a specific subset of cells with considerable immunosuppressive ability (1). As a result, most studies evaluating MDSCs focus on their impact on various pathological conditions, including cancer, chronic infection, sepsis, and autoimmunity (2). MDSCs also play an important role in regulating immune homeostasis under many physiological conditions, and previous research has shown that the accumulation of MDSCs is important for maternal–fetal tolerance *via* their suppression of the T cell response during pregnancy (3). Such studies have focused on the time after implantation of the fertilized egg. Gestational age is calculated from the last menstrual period; thus, it is crucial to investigate how the MDSCs change during the normal menstrual cycle and follicular development. These changes may play a critical role in a normal pregnancy.

Estradiol (E2) levels are markedly increased during pregnancy (3), and MDSC proportions are directly influenced by prostaglandin E2 (PGE2), which regulates vascular endothelial growth factor (VEGF) during *in vitro* fertilization (IVF) (4). E2 levels are also known to be involved in the development of some cancers, with some estrogen receptor α (ERα)-positive stromal cells increasing E2 expression (5), which may promote the progression of cervical and breast cancers in both non-pregnant and pregnant patients *via* its induction of the MDSCs (6). *In vitro*, PGE2 also induces the differentiation and activity of MDSCs (7) by enhancing the suppressive phenotype and functions of GM-CSF/IL-6-induced M-MDSCs (8). However, to our knowledge, there have been no reports describing the relationships between MDSCs, E2, and PGE2 under normal, non-pregnant conditions.

The expression of both E2 and PGE2 experiences cyclic changes in response to the normal menstrual period, with E2 decreasing when the follicles begin to develop and PGE2 increasing substantially prior to ovulation. Given the natural differences in these hormones, we used samples from various points in the menstrual cycle to evaluate their effect on MDSCs and the possible influencing factors in their function under normal conditions. Thus, in this study, we investigated the MDSC ratio during the menstruation (MP) and ovulation (OP) periods and then evaluated the relationship between the associated changes in E2 and PGE2 expression on MDSC differentiation and activation.

## Materials and methods

### Study participants

Ten healthy volunteers were selected as participants. The study was approved by the ethical board at the Qinghai University Hospital (approval number: SL-2020091), and all participants signed informed consent forms. Given that the frequency of MDSCs substantially increases with age (9) and obesity (10), we conducted our study on women with an average age of 30.50 ± 1.51 years and a BMI of 20.15 ± 1.38 kg/m^2^. They all had regular menstrual cycles, none were pregnant, and none suffered from any underlying chronic diseases. In addition, there was no history of drug or hormone use, and all 10 volunteered for this study. The demographic data of these subjects are summarized in Supplementary Table 1.

### Flow cytometry and cell sorting

Myeloid-derived suppressor cell-like cells were collected from the peripheral blood samples of the participants using EDTA anticoagulant tubes. The samples were centrifuged for 20 min at 410 × *g* before the red blood cells were removed using RBC lysis buffer (BioLegend, San Diego, CA, USA, 420301, B337300). The remaining white blood cells were washed twice in phosphate-buffered saline (PBS) and then diluted to a concentration of 1–5 × 10^6^ cells/ml before being evaluated using a BV510-Zombie Aqua Fixable Viability Kit (BioLegend, 77143, B324734), which facilitates live/dead staining, according to the manufacturer’s instructions. We then completed five-color analysis to confirm MDSC identity, including staining for FITC-CD11b (BioLegend, 301330, B272326), BV421-CD33 (BioLegend, 366622, B310764), Percp/cy5.5-HLA-DR (BioLegend, 307630, B293514), PE-CD14 (BioLegend, 367104, B274117), and APC-CD15 (BioLegend, 301908, B270323). Flow cytometry was completed on Aria III (BD Biosciences, Franklin Lakes, NJ, USA), and data were analyzed using the FlowJo software. The Aria III was used for MDSC-like cells sorting, which relied on CD11b^+^HLA-DR^low/–^CD33^+^ identification. The studied cells are referred as MDSC-like cells as they were measured with flow cytometry in whole blood instead of peripheral blood mononuclear cells (PBMCs), and also because of the absence of enough functional assays.

### Estradiol detection and routine blood examination

Standard serum sex hormone detection tests and routine blood analyses were completed by the Laboratory Department at the Qinghai University Affiliated Hospital with each blood sample tested using an XN-2000 (Hisenmeikang, Shanghai, China) complete blood analyzer. Sex hormones were tested using a Cobas 6000 (Roche Diagnostics, Mannheim, Germany) automatic electrochemiluminescence immunoassay analyzer, and all the instruments were calibrated and quality controlled as required prior to testing.

### Prostaglandin estradiol detection

In total, 2 ml of peripheral blood was collected and centrifuged for 20 min at 410 × *g* before the plasma was stored at −80°C. All samples were collected and tested together using the prostaglandin E2 high sensitivity ELISA kit (Abcam, Cambridge, UK, ab133055, GR3347010-5) according to the manufacturer’s instructions. All readings were performed on a microplate spectrophotometer (Bio-Rad Laboratories, Hercules, CA, USA) at 405 nm.

### Arginase activity assay

In total, 30 ml of peripheral blood was collected using EDTA anticoagulant tubes and centrifuged for 20 min at 410 × *g*, after which the white blood cells were separated as previously described. These cells were then stained as described above, and the relevant MDSC-like cells were sorted used the marker CD11b^+^CD33^+^HLA-DR^–^ and saved in arginase activity assay buffer as described by the manufacturer. All samples were collected and tested together using an arginase activity assay kit (Abcam, Cambridge, UK, ab180877, GR3255086-17).

### Statistical analysis

GraphPad Prism version 9.0 was used for all statistical analyses. Data are presented as mean ± SD and were analyzed using paired sample *t*-tests, independent sample *t*-tests, and Pearson correlation tests. A *P-*value of < 0.05 was considered significant.

## Results

### Routine blood examination results of the menstrual and ovulation window samples were similar

To eliminate the heterogeneity within this population and determine whether the differences in MDSCs are associated with other parameters, we first completed a routine examination of these blood samples, comparing the parameters of each subject within the MP and OP. These evaluations revealed no significant differences between the two samples when comparing white blood cell count, neutrophil percentage, neutrophil count, lymphocyte percentage, lymphocyte count, monocyte percentage, monocyte count, hemoglobin, and platelet count (Supplementary Figure 1).

### Estrogen levels decreased while prostaglandin estradiol levels increased in menstrual period samples

**FIGURE 1:**
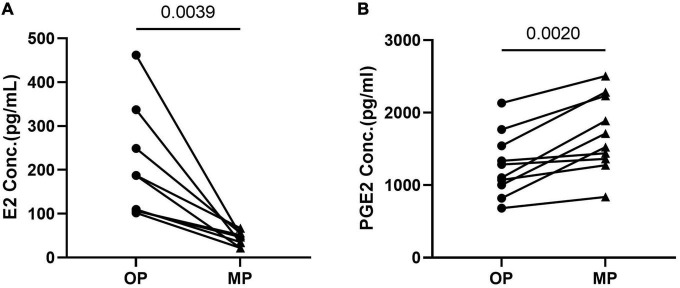
The expression of estradiol (E2) and prostaglandin estradiol (PGE2). **(A)** E2 expression decreased in menstrual period (MP) when compared with ovulation period (OP). *N* = 9. **(B)** PGE2 increased in MP compared with OP. *N* = 10. Significance determined by paired *t*-test *p* < 0.05. The • represents OP and the ▲ represents MP.

We obtained blood samples from every participant during the MP and OP windows of the same menstrual cycle, producing a sample interval of 10–14 days. The greatest differences between the MP and OP samples were the changes in hormone and prostaglandin expression. Given this, we characterized their E2 and PGE2 levels using high sensitivity immunoassays, which revealed that the E2 levels were significantly lower in MP samples than in OP samples (Figure 1A). This is also consistent with the hormonal changes associated with normal menstruation. In addition, our assays showed that the PGE2 levels in the MP samples were significantly higher than those in the OP samples. This is consistent with the endometrium expressing high levels of prostaglandins just before and during the onset of menstruation to aid in the uterine contractions needed to excrete menstrual blood (Figure 1B).

### Percentage of myeloid-derived suppressor cell-like cells in the peripheral blood increased during the menstrual period

**FIGURE 2:**
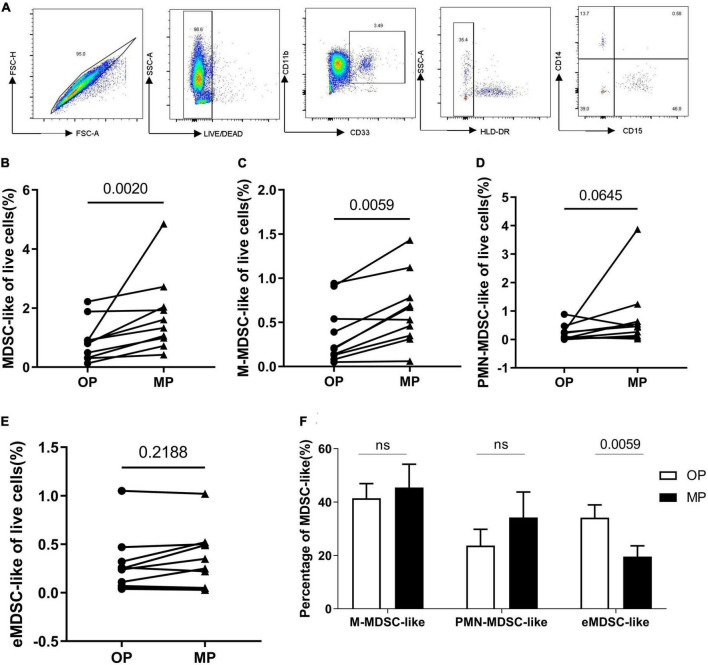
The change of myeloid-derived suppressor-like cells (MDSC-like), granulocytic-MDSC-like (PMN-MDSC-like), monocytic-MDSC-like (M-MDSC-like) and early-stage-MDSC-like (eMDSC-like). **(A)** The gating strategy for MDSC-, PMN- MDSC-, M- MDSC-, and eMDSC-like cells. **(B)** Percentage of live MDSC-like cells increased in the menstrual period (MP) group when compared with the ovulation period (OP) group. **(C)** Percentage of live M-MDSC-like cells increased in MP samples when compared with OP samples. Scatter diagram showing the percentage of **(D)** PMN- and **(E)** eMDSC-like cells within the live cell totals. **(F)** Bar chart showing the percentage of the total MDSC-like cells presenting as M-, PMN- and eMDSC-like cells. *N* = 10. Significance determined by paired *t*-test *p* < 0.05. The • represents OP and the ▲ represents MP.

As mentioned above, there are several different phenotypes of MDSC, each with different functions. Given this, we evaluated the proportion of each of these subsets within the peripheral blood of each participant at both the OP and MP: CD11b^+^CD33^+^HLA-DR^–^ describes the total MDSC-like cells population, CD11b^+^CD33^+^HLA-DR^–^CD14^–^CD15^+^ describes the granulocytic-MDSCs-like (PMN-MDSC-like), CD11b^+^CD33^+^HLA-DR^–^CD14^+^CD15^–^ describes the monocytic-MDSCs-like (M-MDSC-like) (M-MDSCs), and CD11b^+^CD33^+^HLA-DR^–^CD14^–^CD15^–^ describes the early stage MDSCs-like (eMDSC-like) (eMDSC) (11). Each of these populations was identified using six-color flow cytometry (Figure 2A), which revealed a significant increase in the number of total MDSC-like cells as a percentage of the live cells in each MP sample. This increase was particularly marked for M-MDSC-like cells, whereas both the PMN-MDSC-like and eMDSC-like cells numbers did not change (Figures 2B–E). In addition, the proportion of eMDSC-like cells decreased significantly (Figure 2F), although the impact of this remains unclear, as the role of these cells is not yet well understood (11, 12). We speculated that they may act as precursors for PMN- and M-MDSC-like cells.

### Myeloid-derived suppressor cell-like cells counts negatively correlated with lymphocyte percentage, while their arginase activity increased during ovulation period

**FIGURE 3:**
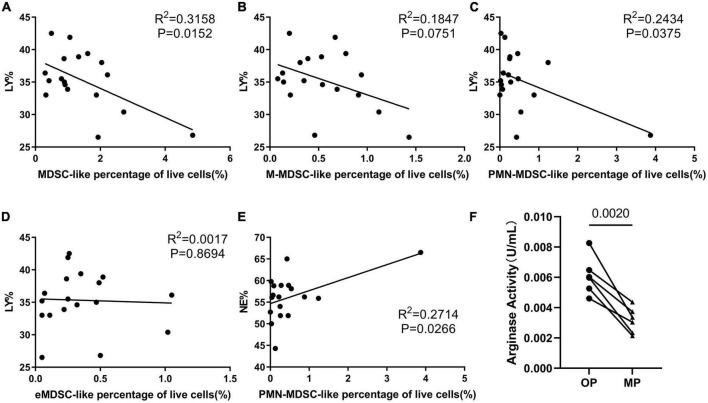
The immunosuppressive ability of myeloid-derived suppressor cell-like (MDSC-like) cells. Correlation analysis evaluating the relationship between several different indices, including correlation between lymphocyte percentage and **(A)** MDSC-like cells percentage, **(B)** monocytic-MDSC-like (M-MDSC-like) cells, **(C)** granulocytic-MDSC-like (PMN-MDSC-like) cells, and **(D)** early-stage-MDSC-like (eMDSC-like) cells. **(E)** Correlation between neutrophil percentage and PMN-MDSC-like cells percentage. **(F)** Arginase activity in the MDSC-like cells from six volunteers in the ovulation period (OP) and menstrual period (MP) groups.

We performed a correlation analysis to evaluate the relationship between MDSC-like cells and other cell types. These evaluations revealed that the percentage of MDSC-like cells was negatively correlated with lymphocyte percentage and that there was a clear reduction in lymphocyte counts with an increase in the number of PMN-MDSC-like cells (Figures 3A–D). Lymphocytes are the most important part of our immune system, and in most cases, our volunteers believed that they were more likely to catch a cold during their menstrual period and develop herpes on their cheeks. Thus, we considered whether this was related to the decreased expression of immune effector cells like the lymphocytes. We also found that the percentage of PMN-MDSC-like cells positively correlated with the percentage of neutrophils (Figure 3E); although this may be because PMN-MDSCs and neutrophils are difficult to distinguish from each other as they share similar surface markers (11, 13). PMN-MDSCs are more difficult to distinguish from neutrophils under physiological conditions than in tumors or chronic inflammation where they express slightly different surface markers. Arginase evaluation revealed an increase in arginase activity in the OP samples, which was not consistent with the MDSC data (Figure 3F).

### Prostaglandin estradiol expression positively correlated with the percentage of myeloid-derived suppressor cell-like cells

**FIGURE 4:**
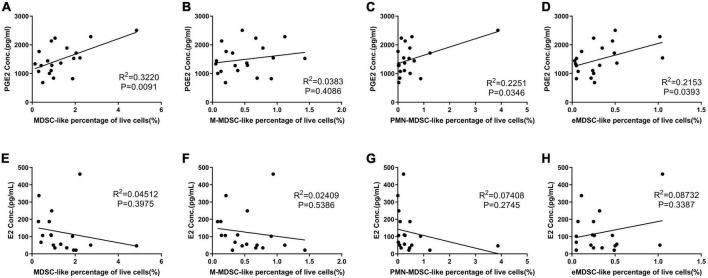
The affect of prostaglandin estradiol (PGE2) and estradiol (E2) on myeloid-derived suppressor cell-like (MDSC-like) cells percentage. **(A–D)** Correlation between PGE2 concentration and MDSC-, monocytic-MDSC (M-MDSC-), granulocytic-MDSC (PMN-MDSC-), and early-stage-MDSC-like (eMDSC-like) cells percentage. **(E–H)** Correlation between E2 concentration and MDSC-, M- MDSC-, PMN- MDSC-, and eMDSC-like cells percentage.

Prostaglandin estradiol plays a critical role in MDSC expansion and maturation (14), as it induces M-MDSC expansion and increases their immune suppression functions (8). Our results showed that the levels of PGE2 were positively correlated with the percentage of MDSC-like cells (Figures 4A–D).

Estrogen receptor α is also expressed on MDSCs (5); additionally, arginase I production is increased when MDSCs are treated with E2, due to activation of the STAT3 pathway *via* this receptor (6). This means that E2 is important in both the expansion and activation of MDSCs during human pregnancy (15). Our results revealed no correlation between E2 and MDSC-like cells counts (Figures 4E–H); however, our OP results suggested a relationship between increased E2 expression and increased arginase activity in the MDSC-like cells isolated in this study.

## Discussion

Menstruation is a marker of sexual maturity in women and involves the functions of many immune cells that synthesize and secrete a large number of inflammatory chemotactic factors, proinflammatory factors, matrix proteinases, prostaglandins, and plasminogen activators that participate in menstrual regulation (16). Menstruation is assumed to be an inflammatory reaction, as the endometrial edema and inflammatory cell infiltration increase the levels of inflammatory factors inducing shedding (17). MDSCs are a group of cells known for their immunosuppressive capacity (1). Therefore, we hypothesized that an increase in the number of MDSC-like cells in the peripheral blood during the MP may be associated with the suppression of overactive immune responses and the maintenance of immune homeostasis. MDSCs are also released following tissue damage caused by trauma or surgery, during which they contribute to tissue repair and the healing process (18). Given this, we suggest that the increase in MDSC-like cells number during the MP may also contribute to endometrial repair, wherein MDSC-like cells may play a role in wound healing (19). As the endometrium is repaired, the inflammatory response is gradually weakened. Therefore, the percentage of MDSC-like cells decreases during ovulation.

A successful pregnancy is facilitated by the egg; thus, it follows that the MDSCs did not disappear in the OP but rather their suppressor activity was increased *via* the increased expression of Arg-1. This change may help to better prepare the sperm for implantation and help to prevent endometrial rejection of the fertilized egg. Arginase activity increased in the OP samples, which was not consistent with the MDSC-like cells data. We propose the following explanations for these differences: (a) during MP, menstrual bleeding initiates bone marrow hematopoiesis, thus facilitating the production of bone marrow-derived MDSCs, whose primary function is to promote tissue repair and wound healing (20, 21); (b) during the OP, the body becomes more conducive to pregnancy (3, 22), which requires an increased level of inflammatory suppression, thus inducing arginase expression in the MDSC-like cells found in these samples. This hypothesis is supported by a previous study that described a positive correlation between increased MDSC numbers and elevated pregnancy rates in IVF patients, independent of the effects of E2 (4); (c) the MDSCs may migrate from the peripheral blood to the endometrium during the OP to facilitate later regulation of the menstrual cycle. All these possibilities need to be validated through further studies.

In this prospective study, we determined the ratio of MDSC-like cells in the peripheral blood of 10 healthy volunteers during the MP and OP. Results showed that the ratio of MDSC-like cells was higher in the MP, while the Arg-1 activity of these MDSC-like cells was higher in the OP, suggesting that MDSCs have a stronger immunosuppressive effect during the OP when the egg is expelled. The increase in this activity may be conducive to the subsequent implantation of the fertilized egg and lays the foundation for a healthy pregnancy. To the best of our knowledge, this is the first study to evaluate MDSCs and E2 and PGE2 expression in normal, non-pregnant patients. Our results showed that (a) the ratio of MDSCs in the peripheral blood changes across the course of the whole menstrual period and (b) these changes help maintain the immune balance and prepare the fertilized eggs for implantation.

Prostaglandin estradiol plays a critical role in MDSC expansion and maturation (15), as it induces M-MDSC expansion and increases their immune suppression (16). We found that the levels of PGE2 were positively correlated with the percentage of MDSC-like cells, which corroborated the important role of PGE2 in MDSC regulation. E2 may promote the progression of cervical and breast cancers in non-pregnant and pregnant patients *via* its induction of MDSCs (6). However, our results showed no correlation between E2 and the percentage of MDSC-like cells in the peripheral blood. This may be because the concentration was still within the normal range, unlike the high expression associated with tumor tissues, but this was consistent with the increased suppressor activity of MDSC-like cells during the OP, in which E2 is more strongly expressed. These results also suggest that E2 may enhance the inhibition of MDSCs.

To adequately understand the dynamic changes in MDSCs during the menstrual cycle, it will be necessary to extend the sampling period to include the other phases of the cycle, such as the follicular and luteal phases. It would also be useful to evaluate these outcomes in patients with infertility or recurrent miscarriage to better support our results.

If we can detect dynamic changes in the MDSC ratios of the peripheral blood, then we can use this information to evaluate immune homeostasis during the MP. This may help to facilitate the early identification of some pregnancy-related diseases, such as infertility and miscarriage.

The small sample size is a limitation of our study, but we believe that this study provides a very new perspective, and it is well worth showing for other researchers.

## Conclusion

There are regular changes in the ratio and capacity of the MDSC-like cells over the course of a normal menstrual cycle. Our results suggest that these MDSC-like cells promote endometrial repair during MP and a permissive immune environment during the OP to promote pregnancy. This change in MDSC-like cells function is related to changes in both E2 and PGE2 expression. These findings may help to better understand the pathophysiology of pregnancy-related complications and lay a foundation for improving perinatal outcomes.

## Data availability statement

The raw data supporting the conclusions of this article will be made available by the authors, without undue reservation.

## Ethics statement

The study was approved by the Ethical Board at the Qinghai University Hospital (approval number: SL-2020091). The patients/participants provided their written informed consent to participate in this study.

## Author contributions

All authors listed have made a substantial, direct, and intellectual contribution to the work, and approved it for publication.
