# Supplementary material for: Dynamic changes in myeloid-derived suppressor cells during the menstrual cycle: A pilot study
Source: Front Med (Lausanne). 2022 Nov 15;9:940554. doi: 10.3389/fmed.2022.940554 (PMC9705596; doi:10.3389/fmed.2022.940554)
Supplement: Supplementary file 1 [file Table_1.DOC]

Supplementary Table 1. Demographic makeup of the study cohort

| Participant  number | Age  (y) | Height  (m) | Weight  (kg) | BMI  (kg/m^2^) | Menstrual Cycle |
| --- | --- | --- | --- | --- | --- |
| 1 | 31 | 1.62 | 55 | 20.96 | 3-5/28 |
| 2 | 30 | 1.62 | 54 | 20.58 | 4/28 |
| 3 | 32 | 1.65 | 57 | 20.94 | 6/28 |
| 4 | 32 | 1.56 | 55 | 22.60 | 5-8/28-32 |
| 5 | 30 | 1.59 | 49 | 19.38 | 4/26 |
| 6 | 31 | 1.60 | 55 | 21.48 | 6-7/28-30 |
| 7 | 28 | 1.60 | 48 | 18.55 | 3-5/28 |
| 8 | 32 | 1.60 | 49 | 19.14 | 4-6/35 |
| 9 | 31 | 1.60 | 50 | 19.53 | 5/28 |
| 10 | 28 | 1.65 | 50 | 18.35 | 6/26-28 |





Supplementary Figure1. The results of routine blood examinations. (A) White blood cell count, (B) percentage neutrophils, (C) neutrophil count, (D) percentage lymphocytes, (E) lymphocyte count, (F) percentage monocytes, (G) monocyte count, (H) hemoglobin and (I) platelet count. N=9, Significance determined by paired t-test, *p* >0.05.
