# Supplementary figures and images for: Dynamic changes in myeloid-derived suppressor cells during the menstrual cycle: A pilot study
Source: Front Med (Lausanne). 2022 Nov 15;9:940554. doi: 10.3389/fmed.2022.940554 (PMC9705596; doi:10.3389/fmed.2022.940554)

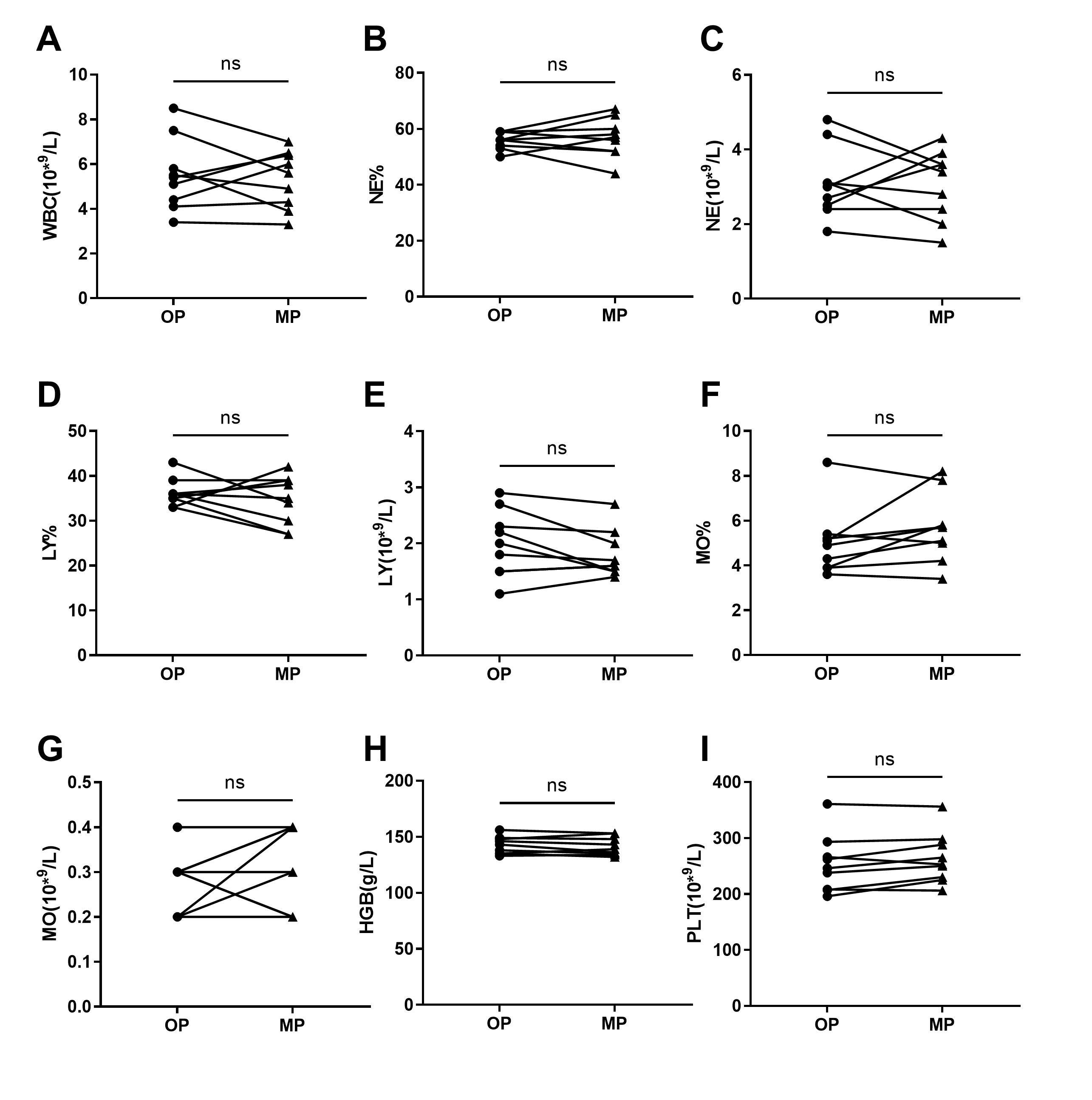

Supplement: Supplementary file 2 [file Image_1.JPEG]
